# Do You Transfer Your Skills? From Sports to Health Management in Cancer Patients

**DOI:** 10.3389/fpsyg.2020.00546

**Published:** 2020-04-29

**Authors:** Valeria Sebri, Lucrezia Savioni, Stefano Triberti, Ilaria Durosini, Ketti Mazzocco, Gabriella Pravettoni

**Affiliations:** ^1^Department of Oncology and Hemato-Oncology, University of Milan, Milan, Italy; ^2^Applied Research Division for Cognitive and Psychological Science, IEO, European Institute of Oncology IRCCS, Milan, Italy

**Keywords:** skill transfer, sport, skills, health management, coach, physician, sport psychologist, subject-centered perspective

## Abstract

Skill transfer is a process where personal cognitive and behavioral abilities are applied to contexts that are different from the one in which they were originally learned. Literature demonstrates that skill transferability is possible: for example, people can apply skills learned in sports to other life-domains (such as school, work, or health management) with the aim to improve individual characteristics and reach personal goals. To do this, several factors, such as positive communication, adequate context, a person-centered perspective, and specific strategies, are necessary. On the basis of this, the aim of this contribution is explore the relationship between sports and health management skills to enhance the coach/athlete as well as the patient/physician relationships. Useful strategies for skill transfer from sports to cancer management are shown.

## Introduction

Skills may be defined as individual characteristics that orient and affect human performances in everyday life activity that can also be trained to reach desirable, if not optimal, levels of functioning (e.g., goal setting, emotional management, and leadership) (Pierce et al., 2017). Several researchers have highlighted that personal skills can be transferred and applied to contexts that are different from the ones in which they were originally learned (Papacharisis et al., 2005; Gould and Carson, 2008; Camiré et al., 2014).

However, how this process of skill transferability can be possible is under discussion (Turnnidge et al., 2014; Allen et al., 2015). First of all, it is not an automatic process, and it is not an outcome or a unique phase either (Danish et al., 2004; Camiré et al., 2012; Martinek and Lee, 2012). In 2017, Pierce, Gould, and Camiré defined the transferability as an ongoing process whereby an individual continually interacts and interprets his/her environments to produce positive or negative skill transfer outcomes. This process can manifest itself in various ways related to the individual’s skills, characteristics, and motivation (Danish et al., 2004). Skill transferability can be easily performed among contexts similar to each other (Leberman and McDonald, 2016) and can be measured, for example, using self-report items (e.g., Aryee and Heng, 1990), questionnaires, or interviews (e.g., Lordly, 2008). For example, people can transfer skills and knowledge from primary school to secondary school. In contrast, when contexts are not similar, people can find it difficult to apply the acquired skills, and they might require higher-order thinking skills, metacognition, and a certain level of cognitive maturity (Martinek and Lee, 2012; Leberman and McDonald, 2016; Jacobs and Wright, 2018). However, even if skill transferability to different contexts is not an instantaneous process, there are significant life-circumstances in which it is essential to use one’s skills to deal with emotions and difficulties (for example, if a person receives a cancer diagnosis, he/she must use their skills to deal with anxiety or sadness) (Faul et al., 2010; Syrjala et al., 2014).

Some researchers have highlighted important steps that can facilitate skill transferability to different life-contexts and help improve certain abilities (Gould and Carson, 2008), such as working on an individuals’ perception of stress management, improving self-efficacy, and developing a personal character (Hardcastle et al., 2015). There are several factors that can improve skill transferability (Allen et al., 2015; Sackett and Gano-Overway, 2017):

(a) value perception of transferable skills
(b) awareness of learned skills and transfer context
(c) confidence in transfer capacity
(d) external support for transfer from significant others
(e) perseverance in case of failure

In particular, people must be aware of the skills learned and, consequently, of the possibility of transferring them (Petitpas et al., 2005).

Regarding the concept of skill transfer from one context to another, sports have a well-known history of being an attractive avenue for helping athletes to develop several skills (Gould et al., 2007; Camiré et al., 2012) and for being a relevant vehicle for the transfer of skills to other life domains (Goudas et al., 2005; Allen et al., 2015; Sebri et al., 2019). From this perspective, Gould and Carson (2008) said that skills are “those internal personal assets, characteristics, and skills such as goal setting, emotional control, self-esteem, and hard work ethic that can be facilitated or developed during sports and are transferred for use in non-sports settings” (p. 60). Skill transfer is important for both individual and team sports (Issurin, 2013). Generally, positive transfer of motor, conceptual, and perceptual variables occurs when sports are similar (Gorman et al., 2011). For example, soccer and rugby, as well as other invasion sports, demand perceptual elements of tracking the ball in flight (Causer and Ford, 2014). Moreover, skill transfer is influenced by the expertise of athletes. Expert athletes have an advantage when it comes to learning and transferring their abilities thanks to daily and continuous training (Moore and Müller, 2014). Nonetheless, the athlete is the main factor that influences skill transfer due to the involvement of his/her internal assets (e.g., demographic data), external assets (e.g., coach and sports psychologist), and previous experience of sports (Pierce et al., 2017) in individual as well as team sports.

From a practical point of view, sports promote cognitive development (e.g., memory, attention, and decision-making), self-perception, and emotions (Mann et al., 2007; Pesce et al., 2009; Pesce et al., 2013). Moreover, athletes learn behaviors and values (Camiré and Trudel, 2010) that are integrated into their own sense of Self by becoming competent in internal, rather than external, behavioral regulation (Deci and Ryan, 2000; Hodge and Lonsdale, 2011). In other words, sports have been considered to be able to promote an overall sense of identity as well as self-esteem (Danish et al., 2004) in accordance with an education “through the physical” and not just “of the physical” (Goudas, 2010). This may be specifically relevant in the field of health management where patients deal with disease and its consequences, as these are also linked to their sense of Self and identity (Zebrack, 2000; Little et al., 2002). Applying skills previously learned to a health management context could be very useful to the pursuit of good treatment results.

## How Can We Transfer Abilities?

Studies have distinguished between two processes to explain how skills can be transferred from one context to another: the implicit and explicit processes (Sackett and Gano-Overway, 2017). In the context of sport, the first process is related to the ability of athletes to transfer acquired skills without intention; the second one is related to a leadership figure, such as the sports coach, or a professional expert, such as the sport psychologist, who intentionally teach abilities and skills with the aim to transfer them in other life domains (Turnnidge et al., 2014; Pierce et al., 2018).

### Implicit and Explicit Processes

The implicit process does not require external support and does not depend on someone’s intention to transfer personal skills (Camiré et al., 2018). Some factors have been highlighted as affecting the implicit transfer of skills: the level of competition, coach–athlete relationship, and peer group interactions, if positive. Conversely, through the explicit process, the coach can intentionally and systematically teach and train abilities (Danish et al., 2004; Papacharisis et al., 2005; Forneris et al., 2012). In order to facilitate transferability, creative environments, guide-driven coaches, or sport psychologists are necessary to develop personal abilities. The first is essential as being conducive to learning thanks to the presence of supportive sport-based relationships (Weiss et al., 2013; Turnnidge et al., 2014); the second one helps individuals to be more aware and confident of the transfer. To achieve these aims, indirect and direct strategies can be implemented (Weiss et al., 2013; Allen et al., 2015). Indirect strategies involve the creation of positive environments (Sackett and Gano-Overway, 2017); direct strategies, instead, consist of skills intentionally implemented through improved cognitive functions and clear rules that allow the transfer (Gould and Carson, 2008; Camiré and Trudel, 2010; Trottier and Robitaille, 2014). As literature says, explicit strategies are more useful to the gain of full use and transfer of skills to other contexts, and this thanks especially to the role of the environment and a guide (Bean and Forneris, 2017; Sackett and Gano-Overway, 2017).

### The Role of the Environment

The environment plays an important role in association with training. It is indeed known that training or education sessions do not bring results if they takes place in an environment that is not suitable for learning (Baker et al., 2003; Gould and Carson, 2008; Camiré et al., 2014). An optimal environment suitable for skill transfer is characterized by attitudes that facilitate participants’ autonomy when making decisions and a feeling of competence when interacting with a sports environment (Sarrazin et al., 2002; Bartholomew et al., 2010; Bengoechea et al., 2017): a context in which effort, self-referenced improvement, and enjoyment are reinforced and where mistakes are seen as valuable instrument for learning (O’Rourke et al., 2013). Duda (2013) defined an “empowering” environment as one that is characterized by a task-involving motivational climate where significant others (such as coaches) are autonomy-supportive and socially supportive. A positive and empowering context is composed of the Five Cs – competence, confidence, connection, character, and caring (Lerner et al., 2005) – with the aim to make athletes free to express themselves. Thus, if a sport coach fosters a positive environment for the athletes and provides good leadership, the sportive setting will be more conducive to the development of life skills (Gould et al., 2007). Only in a context structured for athlete expertise and its specific characteristics can coach and athlete collaborate effectively (Doty, 2006; Voss et al., 2010; Lyras and Peachey, 2011), as these are essential prerequisites for transferring gained abilities from a sports context to a real-world context (Giorgi, 2005).

### Relevant Figures in Sports-to-Health Transferability

People are not always aware of the abilities that are developed during training and, consequently, they do not always have the confidence to employ these skills in other life domains. In this regard, different figures can play an important role.

Generally, from childhood, parents are typically the most influential individuals in children’s lives (Knight et al., 2016). Even in the context of sports, parents in the sampling years have a great and lasting effect on children’s sport involvement (Wylleman et al., 2007). In adult life, the role of the romantic partner also seems to gain importance, influencing the perception of conflict, individual’s eating, and conversation satisfaction (Dailey et al., 2011). The role of coaches can be useful for the ability of athletes to understand learned skills and employ them in other contexts (Petitpas et al., 2005; Camiré and Trudel, 2010; Trudel and Bernard, 2013; Marsollier et al., 2019). To this aim, coaches have to perceive and legitimize their role as being responsible for the positive development of an athlete, which often does not happen (Trottier and Robitaille, 2014; Galatti et al., 2016; Super et al., 2016; Bean and Forneris, 2017). Coaches can reinforce athletes’ motivation and progress, making players active learners who are able to assess situations instead of just passive recipients of training (Denison, 2007; Cushion and Robyn, 2014). In recent years, some programs have been implemented with this objective. One of them is, for example, the Transfer-Ability Programme (TAP), created by Allen et al. (2015), which is a 1-week intervention based on sports conducted at a school. The aim of this sport-based intervention was to make participants aware of seven skills learned in the sporting contexts that are available in the academic environment. These sport-based interventions prove that skill transferability is possible, and coaches have identified five factors that can support this process: pride, opportunities, rewards, support from peers, and transfer experience. Thus, it is important that coaches continually learn and improve their abilities in developing players’ skills in an athlete-centered approach for development (Vella et al., 2011; Trottier and Robitaille, 2014; Hardcastle et al., 2015).

In order to sustain athletes’ holistic development, the sport psychologist is a relevant figure (Friesen and Orlick, 2010). Over the times, sport psychologists have become more and more important in several individual and group sports and may help athletes not only to improve their sport performance but also to improve their quality of living outside of sports (Friesen and Orlick, 2010). Different studies have shown the role of emotions and motivations during sports that can negatively influence athletes’ performances (Di Corrado et al., 2013; Di Corrado, 2017). A sport psychologist can work with athletes to find the right motivations and to learn to manage emotions that could be harmful to the performance and learn to use those that are useful to obtain a peak performance (Martin et al., 2005; Chennaoui et al., 2016). The sport psychologist can also collaborate with coaches to take into consideration not just performances and goals but also athletes’ quality of life (Friesen and Orlick, 2010). Pay attention to athletes’ different personality and dispositional attributes have an influence of their body/mind state for performance (Harmison, 2011) (for example, knowing athletes’ psychological profiles could help them to cope with stress, anxiety, and depression that can emerge during competitions) (Chennaoui et al., 2016).

Some of the available strategies introduced during training could be breathing techniques, imagery/visualization, positive self-talk, and goal setting where the role of a sport psychologist is to assist and support the development of the whole athlete as a person (Bond, 2002). This holistic consultation is important in association with the definition of the individual as a compilation of multiple selves, each with its own needs, roles, and stressors in a continuous and mutual interaction (Friesen and Orlick, 2010). Moreover, sport psychologists can assist coaches to promote their awareness development related to their own personal needs, such as job insecurity, time management, and performance anxiety. Exploring feelings, behaviors, and thoughts becomes the first step for building a working and trust alliance (Giges et al., 2004; Sharp and Hodge, 2013).

## Skills in Sport as Well as in Oncological Health Management

Up until now, we have explored the field of transferability related to essential skills for individual development and improvement in various contexts. Sport in particular is considered to be a special environment in which personal abilities can be learned and transferred. For example, Camiré et al. (2012) conducted a qualitative study and highlighted that coaches and athletes believed that athletes could transfer the skills gained through sports to other life domains.

Based on the themes outlined in this manuscript, our aim has been to deepen understand of the relationship between sports and health management to further explain skill transfer. An interesting field in which to consider the effects of sport–health management transferability is that of cancer.

Tumor diagnosis is a shock experience for patients and their family, and it may elicit overwhelming feelings (Fallowfield, 2008; Fioretti et al., 2016; Triberti et al., 2019b). A cancer diagnosis can elicit experiences of uncertainty linked to existential challenges or changes in lifestyle as well as the everlasting fear of recurrence (Rottmann et al., 2010; Gnagnarella et al., 2016; Triberti et al., 2019a). During and after oncological treatments, physical and cognitive functions are involved. Moreover, cancer interventions, such as chemotherapy, radiotherapy, and hormonotherapy, can produce symptoms of “chemobrain,” which causes cognitive dysfunction in addition to an overall discomfort in terms of mood disorders, convulsions, and vascular complications (Ganz, 2012; Patel et al., 2014). There are indeed consequences to treatment during each phase of disease (prevention, screening, diagnosis, treatment, survival, or end of life) as it makes daily life decisions more complicated in terms of the application of cognitive skills and flexibility (Pardon et al., 2013). However, the management and treatment of a disruptive disease is an occasion for the patient to be engaged in important bonds: within a patient-centered approach, the physician is usually a patient’s safety guide during a condition of uncertainty (Rottmann et al., 2010), and this is similar to the role played by a coach in sports. The doctor is indeed not only an expert consultant but also a point of reference for people at a particularly vulnerable time. Different studies have shown that a good and personal patient–physician relationship is of vital importance to cancer patients (Vogel et al., 2006) for a better therapeutic outcome and shared decision making (Arora, 2003; Barth and Lannen, 2011; Kane et al., 2014; Renzi et al., 2016). The same is true for the coach–athlete relationship: the behaviors of coaches in the form of involvement, autonomy-supportive behaviors, and provision of structure have a beneficial impact on athletes’ needs for autonomy, motivation, and competence (Mageau and Vallerand, 2003). Additionally, communication is a key function of obtaining a desirable relationship with others. In a doctor–patient relationship, both the patient and the doctor become part of a shared experience in which communication becomes the vehicle to exchanging implicit and explicit information, values, and emotions (Roter, 2000). As stated by Epstein et al. (2005), good and efficient patient-centered communication includes the (a) elicitation and understanding of the patient’s perspective (i.e., ideas, concerns, expectations, needs, functioning, and feelings), (b) understanding the patient within his/her psychosocial context, (c) reaching a shared understanding of the disease and the treatment with the patient in line with the patient’s values, and (d) helping patients to share responsibility and power by involving patients in choices. Specifically, it is important that physicians (i) assess what the patient already knows. They should check what a patient knows about his/her condition to see what is already understood or misunderstood. Patients must be able to express their perplexities and preferences (Ishikawa et al., 2013); (ii) assess what patients want/need to know. Patients need to know and understand what is happening and the available treatment options, but not all patients want detailed information (Baxi et al., 2013); (iii) are empathic. They should not ignore or minimize patients’ feelings but should aim to understand and listen. Good communication has to be supportive and empathic to create a cooperative patient–physician relationship in a context perceived as safe (Hall et al., 2010; Kullberg et al., 2015); (iv) slow down and provide information in slow and deliberate fashion in order to give patients the time he/she needs to comprehend the new information. The physician has to use comprehensible language and be thorough as well as pay attention to patients’ life habits, emotions, and fears in order to understand their needs (Pham et al., 2014); (v) keep it simple, using short statements and simple explanations (Morriss et al., 2010); (vi) tell the truth and not minimize the impact of what they are saying (Khalil, 2013); (vii) be hopeful, as the value of conveying hope in some situations should not be underestimated (Werner and Steihaug, 2017); (viii) watch the patient’s body language and facial expressions, paying attention to non-verbal communication (Pawlikowska et al., 2012); and (ix) be prepared for patients’ reactions, as each patient will react to a situation differently (Mazilu et al., 2010).

This way, the comprehension and management of cancer experiences are possible; physicians can use a variety of alternative strategies to facilitate the exchange of information with cancer patients and their families. These methods could also include audiovisual aids, teaching, the Internet, telephone helplines, and written materials. Good information exchange has been recognized as useful to increase patients’ control and involvement in their care by reducing their psychological distress, encouraging better levels of adherence, and instilling realistic expectations (McPherson, 1993). Some sports strategies and skills are therefore useful also for health management, especially in an oncological context where doctor–patient communication foresees a co-construction and activation by both interlocutors. How could the transfer of skills previously learned in sport to oncological contexts be made sustainable?

## What Are the Available Strategies for Skill Transfer?

In accordance with what has been mentioned above, sports could be an excellent field in which to promote skill transferability. Even if people with previous sports experience can show more easily transferable abilities, non-sport people could also profit from sports interventions and enact skill transfer in other contexts. So, how does the transfer process take place? There are some available “strategies” to promote skill transferability from sport to health management through the coach and sports psychologist involvement (see Table 1 below):

**TABLE 1:** Strategies used by coaches to promote skill transfer from sports to health management.

**Strategies**	**Effect on skills**	**What the coach and the psychologist should do**
Willingness of transfer intentionally	Comprehension, confidence, support, and reinforcement in transfer	Organize specific training to support the transfer of value and trust
Keywords, slogan, and examples	Comprehension of the abilities that are transferred and memorize related information	Explain experiences and provide examples
A positive coach–athlete relationship	Activating and reinforcing connections between the sport and the “real-life” experience; creating a positive context that supports human flourishing	Meet athletes’ family, give also positive feedbacks, take into consideration individual differences and needs
Debriefing and peer debriefing	Internalizing abilities learned in athletes’ Sense of Self	Clarify learning outcomes and give meaning to actions
Team imagery sessions	Imagine real-life contexts in which to apply skills	Plan these sessions and encourage players to imaging

1. Focus on the need and willingness to transfer intentionally: individuals must be aware of the value of a specific skill while being willing to transfer it into another context; specifically, in working on comprehension, confidence, and support/reinforcement in transferability, the coach may organize appropriate training in which the value, trust, and strength of the transfer are supported (Camiré et al., 2012).
2. Improving and reinforcing skills beyond sport: coaches may promote the skill transfer, for example, with slogans, keywords, and clear examples from their experience that players can comprehend, keep in mind, and easily apply in other life-domains (Gould and Carson, 2008; Trottier and Robitaille, 2014; Pierce et al., 2017);
3. Creating a positive coach–athlete relationship: putting genuine attention to the athletes’ life beyond sport and emotions over performances (Gould and Carson, 2008; Camiré et al., 2011), by meeting, for example, athletes’ family (Bowers et al., 2014) and providing feedbacks not only when a negative behavior is observed (Olushola et al., 2013; Hardcastle et al., 2015). It is important that the coach considers the athletes beyond their sporting role – as individuals with different needs and characteristics;
4. Debriefing and peer debriefing to make athletes reflect on the process (Hellison, 2011): to help athletes to clarify learning outcomes, coaches have to make actions meaningful in order to facilitate the integration of skills learned through sport into their sense of Self (Berlin et al., 2007; Gilbert, 2011);
5. Having team imagery sessions before or after practices: the coaches have to encourage players to visualize real-life contexts in which they can apply the learned skills (Jacobs and Wright, 2018).

To sum up, previous sport experiences could be an important occasion to easily develop and improve skills that can be applied to health management contexts if needed. In other non-sport cases, the training and application of sport skills is suggested.

## Discussion

With the aim to explore how the transfer of skills is possible, we have shown various factors and available strategies. These elements are involved both in sport and health management contexts because they are essential for promoting abilities and reaching goals. Despite the fact that people who never played sports before could have difficulties in involving themselves in sport activities (Khan et al., 2012), most evidence highlights that playing sports can improve cardiovascular function, aerobic and metabolic fitness, reduce adiposity, and improve postural balance (Oja et al., 2015); in addition, sports can increase self-esteem and social support (Smith et al., 2012), also for people with physical and/or intellectual disabilities (Eime et al., 2013; Richardson et al., 2017). At the same time, possible factors of influence to be engaged in physical activities could be: (1) peer/group support: having other member involved in the same activities in term of strengths and emotional difficulties; (2) being part of a group: especially with the perception of the own group as being important and essential; (3) enjoy activities: being interest and glad to play sports; (4) having transports to reach the gym; and (5) having time to dedicate (Elmagd et al., 2018; Hutzler and Bergman, 2011).

The present work has aimed to focus not just on sports and its benefits, but also on its role as a vehicle of skill transferability. Skills could be recovered and adapted from previous sports experiences to enhance skills in cancer management contexts. Specifically, acquired competencies concerned non-verbal communication (e.g., bodily messages) and the comprehension and management of experiences to deal with in safety and collaborative contexts. In addition, some useful questionnaires may be adjusted to study patients’ profile of mood state in oncological patients as well as in other pathological diseases for improving their quality of life (Di Corrado et al., 2013; Kim and Smith, 2017). There are some examples of methods developed in sport that have then been applied to health and vice versa. For instance, some perceptual-motor techniques are similarly used to optimize rhythmic movements in sports (e.g., Schaffert et al., 2011; Sors et al., 2015) and health contexts (e.g., Pau et al., 2016; Ghai et al., 2018; Murgia et al., 2018; Mezzarobba et al., 2018), for a review, see Schaffert et al. (2011, 2019). Another example is the Profile of Mood States (POMS, McNair et al., 1971; Di Corrado et al., 2014); initially developed in a clinical context, it is now widely used both in health (e.g., Di Corrado et al., 2013; Kim and Smith, 2017) and sports situations (e.g., Di Corrado et al., 2014; Chennaoui et al., 2016; Murgia et al., 2016; Di Corrado, 2017).

Moreover, within training as well as oncological treatments, a multidisciplinary collaboration of professionals is recommended. On one hand, the figure of sport psychologists may be suggested to increase awareness of skills and their application when involving both coaches and athletes (Pain and Harwood, 2004; Gee, 2010); on the other hand, the participation of coaches with specific sport programs during and after cancer treatments can modify parameters in patients’ quality of life, fatigue, and depression by promoting skills (Baumann and Bloch, 2013; Bouillet et al., 2015). It is possible that skill transfer will be more useful in certain phases of illness management. For example, patients may be able to use individual resources and skills in condition of psychological and emotional balance than acute disease conditions. For example, people who are able to maintain emotional balance are better adjusted to use coping skills and specific strategies for handling complex situations (Batool, 2011). Moreover, we have to consider the relevance of health management beyond treatments (e.g., cancer survivors) when people have to deal with other changes after disease to rebuild their daily activities.

## Conclusion

In conclusion, in light of the themes depicted in the present manuscript, we can hypothesize that oncological patients with previous experience of athlete–coach collaboration may be favored in the application of skills because these are learned and employed already. In other cases, the addition of sport/physical activities programs during and after oncological treatments to start working on skills is suggested. The introduction of sport competencies to transfer them to health management is harder but possible, even if people have had no previous sports experiences. This could be very useful in order to promote better treatments. Future studies may develop this hypothesis and its consequences in health domains.

## Author Contributions

VS conceptualized the ideas presented in the manuscript and wrote the first draft. LS contributed with conceptualization and writing. ST supervised the writing and edited the manuscript. ID contributed with substantial revisions to the final version of the manuscript. KM contributed with important intellectual content and final revision. GP contributed with important intellectual content and supervised the whole process.

## Conflict of Interest

The authors declare that the research was conducted in the absence of any commercial or financial relationships that could be construed as a potential conflict of interest.
